# Do older adults benefit from telepsychiatric care: Comparison to younger adults

**DOI:** 10.3389/fpsyt.2022.998401

**Published:** 2022-08-22

**Authors:** Heather G. Belanger, Mirène Winsberg

**Affiliations:** ^1^Brightside Health Inc., Oakland, CA, United States; ^2^Department of Psychiatry and Behavioral Neurosciences, University of South Florida, Tampa, FL, United States

**Keywords:** telehealth, depression, outcome, telepsychiatry, older adults

## Abstract

**Background:**

Telemental health platforms may increase access to care for older adults. Historically, older adults have tended to adopt new technologies at a slower rate which creates a perception that they may not be able to benefit from them. The purpose of this study was to determine whether or not older adult patients receiving psychiatric care for depression *via* a telemental health platform achieve the same outcomes as younger adults.

**Method:**

Participant data utilized in the current investigation were obtained from a national mental health telehealth company (i.e., Brightside) and consisted of 12,908 U.S.-based adult patients receiving psychiatric care for depression between October, 2018 and January, 2022. Propensity matching was used to create an older and younger sample (n = 141 in each) using 23 covariates. These samples were then compared using repeated measures ANOVA on Patient Health Questionnaire-9 (PHQ-9) scores at start of treatment, 6 weeks, 8 weeks, 10 weeks, 12 weeks, 14 weeks, and 16 weeks.

**Results:**

Despite matching, the groups still significantly differed on prior mental health treatment, such that more older adults reported having had prior mental health treatment. There were no other differences between the groups on assessed variables. Both younger and older adults had decreasing scores over time with no significant differences between them.

**Conclusion:**

Older adults have similar improvement in depression symptom severity over time following initiation of psychiatric treatment *via* a telehealth platform. These findings suggest that age is not a barrier to benefitting from telepsychiatric care.

## Introduction

Major depressive disorder (MDD) is one of the most prevalent (1) and impactful health disorders in the country. It is one of the leading causes of disability in the United States, though only about 65% of people suffering from depression receive treatment (2). Barriers to care may include lack of health insurance/money, limited availability of providers/access, transportation challenges, stigma, and distress associated with having a psychological impairment (3–5). For older adults, these barriers may be compounded by cognitive and/or sensory deficits, social isolation, and physical illnesses.

While evidence is mounting that digital mental health care options can help eliminate structural barriers to evidence-based care (6–16) there remain concerns that older adults may not benefit from telehealth platforms to the same extent as younger adults due to discomfort with technology, cognitive or sensory issues, etc. In general, older adults report less comfort and efficacy with computers than younger adults (17). Particularly during the COVID-19 pandemic, telemental health services have seen extraordinary popularity and growth (18). Questions remain, however, about whether or not older adults, given potentially lesser comfort with technology, will benefit to the same degree.

There is often an assumption that older adults may have more negative attitudes toward telemental health interventions. Indeed, when asked about preferred mode of treatment immediately following stay-at-home restrictions due to COVID-19, those over age 45 were significantly more likely to choose the telephone over video modalities (19). However, survey data suggest that once they use technology for mental health treatment, older adults' satisfaction is high, and no different from in person treatment (20–23). Importantly, telepsychiatry services reduce driving and wait time (21), no-show rates (24), and may reduce overall cost (25). Reviews of the literature have concluded that the vast majority of patients and healthcare providers are satisfied with telepsychiatry services (23, 26, 27), though challenges with technology and training continue to raise concerns (28).

The goal of the current study is to determine whether or not older adult patients being treated for depression *via* a telemental health platform achieve the same outcomes as younger adults.

## Methods

### Participants

Participant data utilized in the current investigation were obtained from a national mental health telehealth company (i.e., Brightside) and consisted of 12,908 U.S.-based adult patients, aged 18 to 82 (mean age = 32.81, sd = 8.92) receiving psychiatric care for depression between October, 2018 and January, 2022. Participants were eligible if they (a) were diagnosed with Major Depressive Disorder by their provider (b) had moderate to severe symptom severity at intake (PHQ-9 ≥ 10) (c) were prescribed at least one psychiatric medication (described below), and (d) had complete outcome data. Patients at high risk for suicide, and patients with psychosis or in need of emergency psychiatric services at the initial evaluation were not eligible.

### Procedure

All study procedures were approved by the WCG Institutional Review Board for the retrospective analysis of patient data obtained by Brightside as part of routine clinical care. Enrolled Brightside patients complete an initial digital intake that includes clinically validated measures of depression and anxiety, as well as questions about clinical presentation, medical history, and demographics. All Brightside patients are required to complete baseline and intake questionnaires. During a patient's first session, a licensed professional prescribed psychiatric medication(s) for each patient. Over the course of treatment, patients communicate with their provider both asynchronously *via* messaging and synchronously *via* video telehealth sessions. Brightside also uses a measurement-based approach to tracking long-term outcomes by prompting patients to complete periodic assessments during treatment. Assessments were completed at baseline/intake, and periodically thereafter. Surveys were administered digitally through an email prompt. Survey completion at baseline, 6 weeks, 8 weeks, 10 weeks, 12 weeks, 14 weeks, and 16 weeks were required for participation.

### Measures

The Patient Health Questionnaire-9 (PHQ-9) is a 9-item self-report measure used to assess the severity of depressive symptoms present within the prior 2-weeks as outlined by DSM-5 criteria. Respondents rate items on a 4-point Likert scale [0–3] and total scores range from 0–27, with >9 indicating mild-to-low symptoms and 10 + indicating moderate-to-severe symptoms. (29) The PHQ-9 shows strong reliability, demonstrating 88% sensitivity and 88% specificity for Major Depressive Disorder (29). There is also evidence that the PHQ-9 can be used as a measure of antidepressant response (30). PHQ-9 scores were collected *via* self-report electronically at baseline, and at weeks 6, 8, 10, 12, 14, and 16 and served as the outcome measure of interest. As part of the PHQ-9, patients were asked to what extent, if they scored >0, these problems have made it difficult for them in four areas—social, family, work, and activities, on a scale from 0 to 3, with 0 indicated “not difficult at all”, 1—“somewhat difficult”, 2—“very difficult” and 3—“extremely difficult” (31). These were summed to create a measure of the functional impact of depression (31).

The GAD-7 is a 7-item self-report measure of Generalized Anxiety Disorder (GAD) symptoms with a four-point Likert scale and a total score ranging from 0–21. Like the PHQ-9, a higher score corresponds to a greater anxiety severity. The GAD-7 has good psychometric properties with 89% sensitivity and 82% specificity for GAD (32, 33).

Other standard demographic and health information was also collected at baseline, such as age, sex, education, race/ethnicity, employment status, income, prior episodes of depression (none, one, or more than one), duration of the current episode, and total number of chronic health conditions endorsed (including arrhythmia, asthma, cancer. hypercholesterolemia, diabetes, heart condition, irritable bowel syndrome or Crohn's disease, lung disease, obesity, thyroid disease, eating disorder, and chronic pain/fibromyalgia).

### Interventions

Because this is a naturalistic sample, participants were prescribed a variety of medications. The most commonly prescribed medication category of the sample (63.7%) was selective serotonin reuptake inhibitors (SSRIs), followed by norepinephrine and dopamine reuptake inhibitors (NDRIs, 19.5%), serotonin-norepinephrine reuptake inhibitor (SNRI, 5.5%), trazodone (or trazodone + SSRI) (4.2%), SSRI and NDRI combination (3.9%), mirtazapine (or mirtazapine + SSRI) (1.8%), and atypical antipsychotics (1.5%). The dosage of index antidepressants remained relatively consistent throughout the study period and were prescribed in standard therapeutic ranges. Dosage adjustments were made based on participant responses to the PHQ-9 and other assessments, as well as virtual visits between participation and providers. Because specifics about treatment were not the focus of this study and because this was a naturalistic study, medications and dosages were not controlled and therefore varied to meet individual needs. 19.5% of the sample was concurrently engaged in psychotherapy.

### Data analyses

Data analyses were performed *via* SPSS, Version 28. Two age-defined groups were created, one group with ages below 60 and one group with ages above 60. Comparisons between groups were made using *t*-tests for continuous variables and chi-square analyses for categorical and evaluated at *p* < 0.01. Propensity-matching of the two groups using 0.001 caliper, was done based on a priori variables collected at baseline that might potentially affect outcome (34). This approach attempts to replicate a randomized trial by obtaining treatment groups with similar distributions of known covariates (35). Included variables were: sex, race/ethnicity, education level, employment status, income level, census-defined region of the country, primary non-mood symptom complaint (agitation, concentration, motivation, sleep, none), past/present use of antidepressant medication, history of any prior mental health treatment, total number of chronic medical conditions (arrhythmia, asthma, cancer, hypercholesterolemia, chronic pain, diabetes, fibromyalgia, heart condition, irritable bowel syndrome/Crohn's disease, lung disease, thyroid disease, obesity), current smoker, prior depression (yes/no), duration of depression, baseline depression and anxiety symptom severity, functional impact of depression rating at baseline, frequency of social media use from 0 to 4 (i.e., never, rarely, several times/week, once/day, several times/day), current participation in concurrent psychotherapy, and frequency of technology use on a scale from 0 to 4 for personal (non-work) use (e.g., phone, tablet, computer, gaming console). Repeated measures analysis of variance (ANOVA) was used to compare the groups over time (at baseline, and at weeks 6, 8, 10, 12, 14, and 16) on total PHQ-9 scores over time. Mauchly's test was used to test the sphericity assumption, with the Greenhouse–Geisser correction (36) used for violations.

## Results

In the entire sample, there were 12,740 individuals in the 18- to 59-year-old age group and 168 in the 60 to 82 year old age group. Besides age, these groups differed significantly on several variables. The older group had significantly fewer people fully employed, χ^2^ = 81.12, *p* < 0.001, had more people in the highest income level, χ^2^ = 48.60, *p* < 0.001, greater number of graduate degrees, χ^2^ = 22.70, *p* < 0.001, greater number of chronic medical conditions, *t* = 12.72, *p* < 0.001, more who had had one prior depressive episode, but fewer who have had several, χ^2^ = 34.86, *p*<.001, more who were currently engaged in both medication and psychotherapy treatment, χ^2^ = 27.58, *p* < 0.001, more who had had prior mental health treatment, χ^2^ = 21.17, *p* < 0.001, less perceived functional impact of depression severity on everyday life, *t* = 4.10, *p* < 0.001, and less anxiety at baseline (as measured by GAD-7: older adults mean = 12.88, younger adults mean = 14.84), *t* = 5.53, *p* < 0.001.They did not significantly differ on race/ethnicity, sex, region of the country, duration of depression, smoking, baseline depression severity (on PHQ-9), technology or social media use, or endorsement of sleeping difficulties, low energy/motivation, agitation/irritability, or difficulty concentrating. Please see Table 1 for a summary of the initial sample. A repeated measures analysis of variance (ANOVA) comparing the younger and older groups on depression severity across time revealed that PHQ-9 scores differed significantly across time, F = 583.19, *p* < 0.000, η^2^ =.069, such that scores significantly decreased over time. There was no group x time interaction, *F* = 0.04, *p* = 0.85, η^2^ = 0.000, meaning the groups both had decreasing scores over time with no significant differences between them.

**Table 1:** Characteristics of younger and older adults, entire sample (*N* = 12,908).

**Characteristic**	**Younger adults**	**Older adults**	***t*** **or χ^2^**	**Effect size^a^**	***p*** **-value**
Age	33.40 (8.20)	64.24 (4.27)	50.25	8.16	<0.001
**Sex**					
Male	31%	33%	0.43	0.01	0.513
Female	69%	67%			
**Education:**					
No high school	1	2%	22.70	0.04	<0.001
High school diploma	31%	23%			
Some college	14%	15%			
College degree	37%	30%			
Graduate degree	17%	30%			
**Race/ethnicity**					
White/caucasian	78%	90%	16.52	0.04	0.01
Asian	48%	1%			
Hispanic	8%	4%			
Black/african american	4%	3%			
Other	6%	2%			
**Employed**					
Full time	69%	41%	81.12	0.08	<0.001
Part time	11%	12%			
Unemployed	20%	47%			
**Annual income**					
<$30,000	30%	14%	48.60	0.06	<0.001
$30–60,000	31%	27%			
$60–100,000	21%	22%			
>$100,000	18%	37%			
**Region of the country**					
Midwest	16%	15%	2.33	0.01	0.51
Northeast	19%	16%			
South	38%	39%			
West	27%	30%			
**Prior episodes of depression**					
None	38%	46%	34.86	0.05	<0.001
One	11%	23%			
More than one	51%	31%			
Prior mental health Treatment	11%	33%	21.17	0.27	<0.001
Number of chronic medical conditions	0.57 (0.85)	1.42 (1.36)	12.72	0.86	<0.001
Baseline PHQ-9	18.17 (4.29)	17.58 (4.25)	1.79	4.29	0.074
Baseline GAD-7	14.84 (4.58)	12.88 (4.89)	5.53	4.58	<0.001
Functional impact total	9.70 (2.01)	9.06 (2.58)	4.10	2.02	<0.001
**How long depressed**					
<2 weeks	1%	2%	9.43	0.03	0.06
2 weeks to 2 months	12%	13%			
2 months to 1 year	27%	35%			
1 to 2 years	17%	17%			
More than 2 years	43%	33%			
**Primary non-mood symptom**					
Sleep	14%	9%	1.27	0.07	0.26
Motivation/low energy	40%	31%	2.63	0.09	0.11
Agitation/irritability	7%	8%	0.05	0.01	0.82
Concentration	3%	6%	0.73	0.05	0.39
Current smoker	10%	10%	0.00	0.00	1.00
**Current treatment**					
Medication	96%	73%	27.58	0.31	<0.001
Medication + therapy	4%	27%			
**Frequency of technology Use, 0–4**					
Seldom, never	5%	8%	4.87	0.13	0.30
Rarely	25%	16%			
Few times/week	25%	28%			
Once/day	18%	16%			
Multiple times/day	27%	32%			
**Social media use, 0–4**					
Seldom, never	16	21%			
Rarely	31%	23%			
Few times/week	15%	14%			
Once/day	32%	28%			
Multiple times/day	6%	13%			

a Effect sizes are Cohen's d for continuous variables and Cramer's V for categorical variables. Effect sizes are interpreted as small (0.2), medium (0.5), and large (0.8). Cohen (37) Mean values are presented for continuous variables (with standard deviations in parentheses) and frequency counts are presented (with %) for categorical variables.

**Figure 1:**
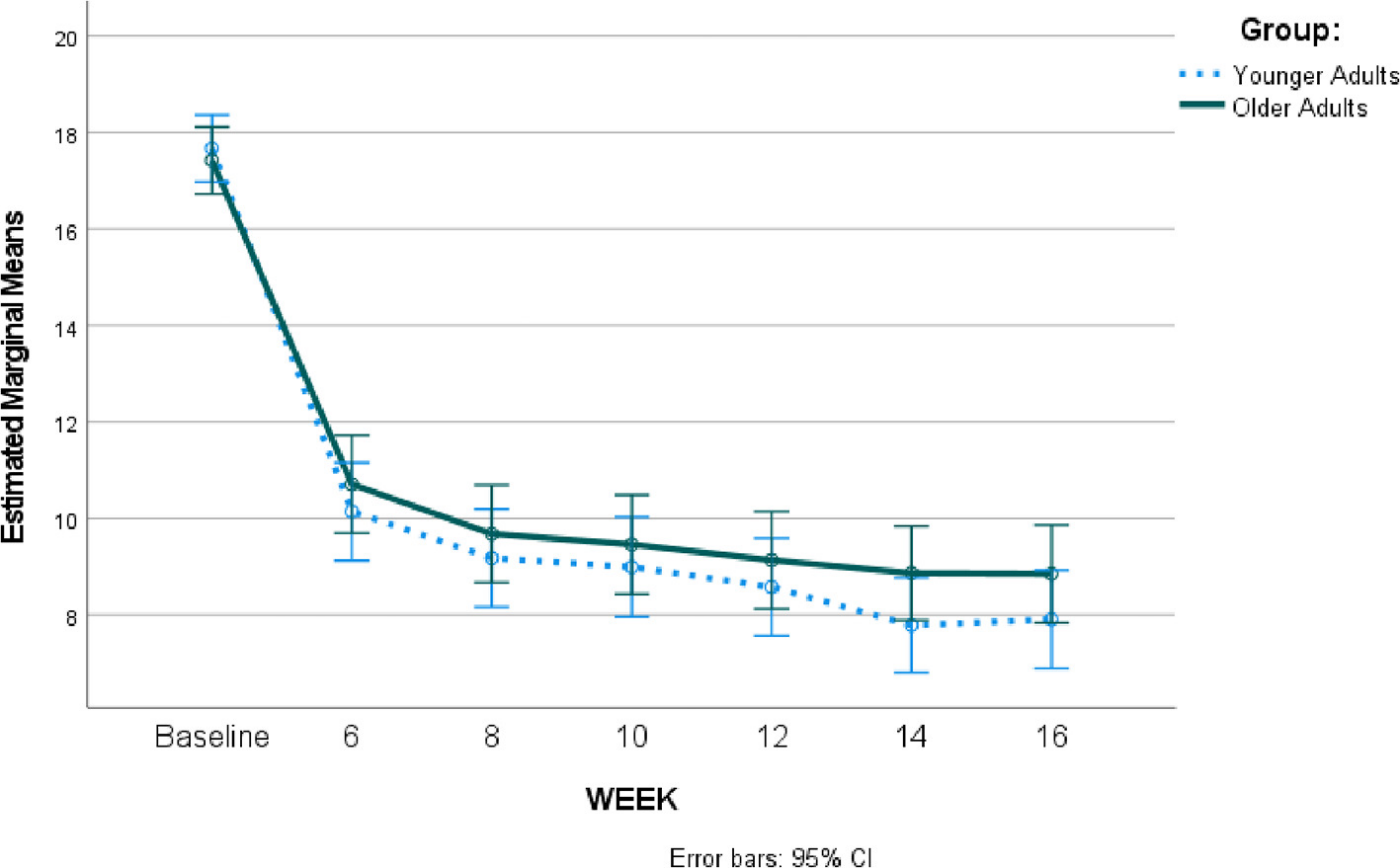
Repeated measures results comparing young vs adults depression severity over time during telepsychiatry treatment.

Due to the differences between groups at baseline, propensity matching was used to create matched groups with 141 in each group. Despite matching, the groups still significantly differed on prior mental health treatment, χ^2^ = 21.17, *p* < 0.001, such that more older adults reported having had prior mental health treatment. There were no other differences between the groups on assessed variables. Repeated measures ANOVA comparing the younger and older groups on depression severity across time revealed that PHQ-9 scores differed significantly across time, F = 263.65, *p* <.001, η^2^ = 0.617, such that scores significantly decreased over time. There was no group x time interaction, *F* = 1.11, *p* = 0.36, η^2^ = 0.004, meaning the groups both had decreasing scores over time with no significant differences between them. Please see Figure 1 for these results.

## Discussion

This study demonstrates that older adults using a completely virtual modality achieve similar outcomes in depression severity as younger adults. This was true in both matched and unmatched samples. Older and younger adults made similar progress over time with telepsychiatric care. There can be an assumption that older adults do not want to use technology and indeed they have historically been slower to adopt new technologies (38). However, in this sample, there were no significant differences between younger and older adults on technology or social media use.

Of course, this is a group of individuals who chose a telemental health platform for their mental health care, so this particular group of older adults is a select sample. However, a more recent study suggests eagerness to adopt and use technology by older adults, as least for tablet use (39). This may be due to the pandemic and the increasing use of technology by society in general during this unprecedented event. Engagement with telehealth may be more a factor of socioeconomic status (i.e., broadband access) than age (40). Even if older adults prefer “in person“ care (not addressed by this study), the results of this study suggest that they can nonetheless benefit from them. It's also important to note that use of technology among older adults tends to increase, and attitudes improve, with use of and training in video technology (41).

These results are like those of a much smaller study that found that psychotherapy delivered digitally was feasible with 20 older adults, and resulted in significantly reduced depression severity after 8 weeks (42). They are also consistent with studies done in the larger healthcare setting, suggesting that telehealth interventions for health-related outcomes (i.e., body weight, blood pressure, activity level, fatigue, etc.,) are effective in older adults (43).

Limitations of this study include selection bias, such that results may not apply to all older adults. Conceivably those who opt into treatment by a telemental health provider are inherently more comfortable with technology and may therefore be in a better position to benefit from it. Also, 90% of the older adults in this sample were white, which again suggests selection bias and speaks to potential digital inequality and white privilege. The results, as such, may be limited to this select group. In addition, the lack of significant difference does not mean that there is not one, though the partial eta squared value (0.004) suggests that an inordinately large sample size would be needed to reveal such a small effect. Finally, this study lacked a control condition not receiving care, preventing any comparative conclusions regarding the effect of treatment.

## Data availability statement

The original contributions presented in the study are included in the article/supplementary materials, further inquiries can be directed to the corresponding author.

## Ethics statement

The studies involving human participants were reviewed and approved by WCG IRB. Written informed consent for participation was not required for this study in accordance with the national legislation and the institutional requirements.

## Author contributions

MW contributed to the conceptualization and writing of the manuscript. HB contributed to the coneptualization, writing, and conducted the analyses. All authors contributed to the article and approved the submitted version.

## Conflict of interest

Authors HB and MW were employed by Brightside Health Inc.

## Publisher's note

All claims expressed in this article are solely those of the authors and do not necessarily represent those of their affiliated organizations, or those of the publisher, the editors and the reviewers. Any product that may be evaluated in this article, or claim that may be made by its manufacturer, is not guaranteed or endorsed by the publisher.
